# Rapid and efficient localization of depth electrodes and cortical labeling using free and open source medical software in epilepsy surgery candidates

**DOI:** 10.3389/fnins.2013.00260

**Published:** 2013-12-31

**Authors:** Juan Pablo Princich, Demian Wassermann, Facundo Latini, Silvia Oddo, Alejandro Omar Blenkmann, Gustavo Seifer, Silvia Kochen

**Affiliations:** ^1^Epilepsy Section, Neurosciences Clinic and Applicated Center, Hospital Ramos Mejia, Universidad de Buenos AiresBuenos Aires, Argentina; ^2^Fundación Favaloro, Resonancia Magnética, NeuroimágenesBuenos Aires, Argentina; ^3^Imágenes Médicas –Neuroimágenes, Resonancia Magnética, Hospital de Pediatría SAMIC Prof. Dr. Juan Pedro GarrahanBuenos Aires, Argentina; ^4^Department of Radiology, Harvard Medical School, Brigham and Women's HospitalBoston, MA, USA

**Keywords:** epilepsy, electrodes, seeg, MRI, localization

## Abstract

Depth intracranial electrodes (IEs) placement is one of the most used procedures to identify the epileptogenic zone (EZ) in surgical treatment of drug resistant epilepsy patients, about 20–30% of this population. IEs localization is therefore a critical issue defining the EZ and its relation with eloquent functional areas. That information is then used to target the resective surgery and has great potential to affect outcome. We designed a methodological procedure intended to avoid the need for highly specialized medical resources and reduce time to identify the anatomical location of IEs, during the first instances of intracranial EEG recordings. This workflow is based on established open source software; 3D Slicer and Freesurfer that uses MRI and Post-implant CT fusion for the localization of IEs and its relation with automatic labeled surrounding cortex. To test this hypothesis we assessed the time elapsed between the surgical implantation process and the final anatomical localization of IEs by means of our proposed method compared against traditional visual analysis of raw post-implant imaging in two groups of patients. All IEs were identified in the first 24 H (6–24 H) of implantation using our method in 4 patients of the first group. For the control group; all IEs were identified by experts with an overall time range of 36 h to 3 days using traditional visual analysis. It included (7 patients), 3 patients implanted with IEs and the same 4 patients from the first group. Time to localization was restrained in this group by the specialized personnel and the image quality available. To validate our method; we trained two inexperienced operators to assess the position of IEs contacts on four patients (5 IEs) using the proposed method. We quantified the discrepancies between operators and we also assessed the efficiency of our method to define the EZ comparing the findings against the results of traditional analysis.

## Introduction

Depth intracranial electrodes (IEs) placement is one of the most used procedures to identify the epileptogenic zone (EZ) in surgical treatment of drug resistant epilepsy patients, about 20–30% of this population (Rosenow and Lüders, 2001). Since the 1950s, IEs recordings have been performed using multiple contact electrodes placed according to Talairach's stereotactic method (Talairach et al., 1992). Electrode positioning is established on each patient based upon hypotheses about the localization of the EZ and electrical spread pathways (McGonigal et al., 2007). Accurate interpretation of the ictal origin of intracranial EEG signal is usually sufficient to define the EZ when concordant with the patient habitual ictal semiology. The EZ is elicited by IEs recordings during a spontaneous seizure targeting the resective surgery. Functional mapping using electrodes stimulation is also performed to define eloquent cortex and to prevent post-operative functional deficits. That information is essential for taking final treatment decisions and depends greatly on the precise localization of IEs.

The anatomical localization of IEs after implantation in early stages of the EEG recording, is a critical issue for the interpretation of neurophysiologic results and surgical planning that has great potential to affect outcome (Gonzalez-Martinez et al., 2012). It allows neurophysiologist to confirm or reject hypothesis about the definition of the EZ and electrical spread pathways. Negligent conclusions are thus avoided discriminating spurious signal and artifacts based on IE's position. Inappropriate physiologic and anatomical assumptions during precocious EEG recording may perpetuate misinterpretations and become a common source of error defining the EZ.

Immediate access to that information is important not only for clinical decisions, but also for the design and implementation of different research programs including tests for cognitive function, evoked potentials, functional connectivity and for single cell recording, in order to achieve more reliable findings.

The method of visual identification is traditionally used by experts to localize IEs on post-implant MRI or CT during chronic implantation in epilepsy. This requires costly resources and specialized multidisciplinary teams to work synchronously. Anatomical localization of IEs is time consuming and the interpretation of results is constrained by the availability of qualified personnel registering the EEG signal.

We describe a methodological process to identify the anatomical location of depth electrode arrays after implantation.

Our method uses established free and open source medical image computing platforms for biomedical research: (3D) Slicer and Freesurfer. 3D Slicer enables the fusion of pre-implant MRI and post-implant CT and Freesurfer produces an anatomical parcellation of the cortex. Particularly we take advantage of these capacities to localize the IEs and their relation with the surrounding cortical structures during the first instances of intracranial EEG recordings.

This approach is intended to reduce time and avoid the need for highly specialized medical human resources to identify IEs.

In order to test this hypothesis we assessed the time elapsed between the surgical implantation process and the final anatomical localization of IEs by means of our proposed method compared with that made based on traditional visual analysis of raw post-implant imaging.

To validate our results; we trained two inexperienced operators to assess the position of 5 individual contacts on four patients using the proposed method. We quantified the discrepancies between operators and we also assessed the efficiency of our method to define the EZ comparing the findings against the results of traditional visual analysis made by experts.

## Materials and methods

### Section 1

Two groups of patients implanted exclusively with depth IEs (AdTech TM, WI, USA) were analyzed. We compared time from implantation to the IE's identification using the proposed method against the traditional visual analysis in a control group. The number of electrode contacts in each depth electrode array was 5–10. The inter-electrode spacing was 5 mm, with 1 mm of cylindrical diameter, and 2.4 mm in length in all cases. All mentions to IEs make reference to an entire intracranial electrode array or group of electrodes; each depth electrode may contain multiple individual contacts. Contacts within an electrode are usually identified with continuous and arbitrary numbers beginning from the deep (contact number 1, corresponding to the tip) to the base (last contact, number 5–10) in the surface. Identification of individual contacts from an IE array is important to define more precisely the anatomical region that is being sampled. The use of depth electrodes, grids or strips is part of our common clinical diagnosis protocol for the surgical treatment of resistant epilepsy patients (Kochen et al., 2002). This protocol may involve the use of invasive explorations with IEs. This study was approved by the research ethics committee of the Ramos Mejía Hospital. Patients gave their informed consent accepting the procedures used in this study and the use of the information.

The first group included four patients; 1 male, median age 29, 5 years (29–37 years) that were processed according to our proposed method and accomplished the required imaging characteristics. They were studied in the last year, and ultimately underwent surgical treatment.

Imaging parameters, pre-processing, registration and visualization practice were standardized and are described below in section 2.

The control group was assessed using traditional visual analysis of raw post-implant CT and MRI. This group included seven patients; the same 4 previous patients plus other three median age 28 years (20–42 years) 3 males; that were ineligible to be processed following our proposed mode due to inappropriate imaging resolution; according to different off-site referral centers.

Demographic and epilepsy characteristics, including age; gender; age at onset; seizures frequency; epilepsy evolution time; medical treatment response; and generalization rates, along with clinical presentation and implantation procedures did not significantly differ between groups. (See Table 1).

**Table 1:** **Demographic data**.

	**Proposed method (4)**	**Control group (3)**	***p***
Age years, median (UQ–LQ)	29.5 (37–29)	28 (42–20)	0.3^*^
Sex male (%)	1 (25%)	3 (100%)	0.14^**^
Age of onset, median (UQ–LQ)	9 (13–5)	4 (6–3)	0.4^*^
Right handed (%)	4 (100%)	3 (100%)	–
Years with epilepsy, median (UQ–LQ)	24 (27.5–20.5)	24 (39–14)	0.85^*^
MRI lesion (%)	2 (50%)	2 (66%)	0.7^**^
Relevant past medical history (%)	1 (25%)	0	–
Seizure frequency per week, median (UQ–LQ)	5 (7–2)	4 (9–3)	0.8^*^
Drug resistant epilepsy (%)^****^	4 (100%)	3 (100%)	–
Number of AEDs received, median (UQ–LQ)	7.5 (9.5–6)	7 (7–5)	0.57^*^
Generalization rate, mean (*SD*)	0.27 (0.15)	0.38 (0.53)	0.7^***^

* Mann Whitney U.

** Fisher test.

*** One way ANOVA.

**** Kwan and Brodie, 2010. AEDs, Anti-epileptic drugs.

The traditional analysis was performed by our team of expert epileptologists, neuro-radiologists, bio-engineers and neurosurgeons with more than 5 years of experience in the field. The IE's were identified by the experts on each patient based on post-implant imaging. IEs were visually inspected on multi-planar reconstructions of post-implant volumetric MRI. The position of IEs was assessed each time by the existing qualified personnel based on their anatomical knowledge. IEs trajectory was identified on MRI as signal voids related to metallic local distortions. Post-implant CT provided accurate image of the electrodes position based on its high Hounsfield units in difficult cases when MR images were uncertain.

The proposed method involved a fusion process between pre-implant MRI and post-implant CT of each patient. Then we aligned the images containing the IEs with an automatic parcellation of the brain to define the anatomical localization of each contact. To clarify the implantation process; 3D pial surface reconstructions were overlaid with the IEs.

Finally, to validate our method; we trained two independent fellows in neurophysiology without any previous experience using the software for a period of about 8 hs. Both operators; blinded to the clinical history and implantation planning were then instructed to determine the anatomical location of 5 individual contacts on different IE's. These contacts were related to the ictal-onset zone in 4 patients of the first group (Two ictal-onset contacts were defined for Patient 2).

The anatomical regions determined by inexperienced operators using our method were compared with those of traditional analysis. Thus the location of contacts was previously established by experts using traditional visual analysis; and considered as a reference standard. Each anatomical region containing these contacts was assumed to include the EZ based on neurophysiologic and surgical results. All regions were ultimately included in the surgical resection.

The validation process was performed two times on different sessions by each trained operator.

We calculated inter-observer variability based on the percent of agreement between operators defining the anatomical cortical region for each 5 contacts. Intra-observer reliability was assessed for each operator comparing the discrepancies between the two sessions.

The accuracy of the process was calculated based on the percent of agreement between the anatomical IEs localization made by inexperienced operators compared to the results of experts using traditional analysis.

**Figure 1:**
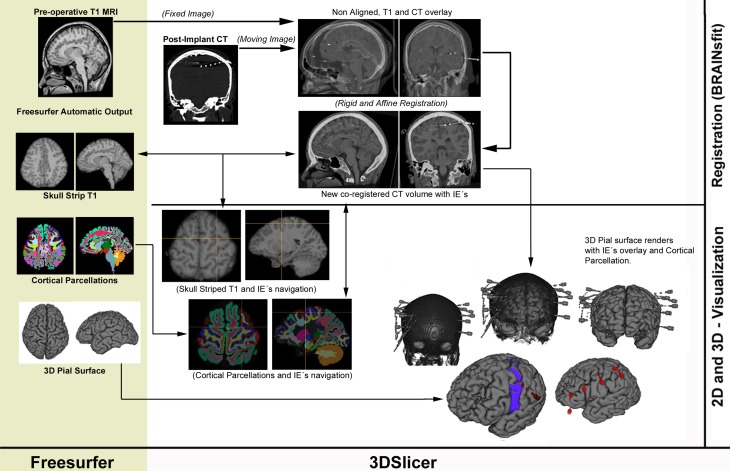
**Proposed workflow pipeline describing Freesurfer's ouput (left column) and the registration/visualization steps performed in 3DSlicer (right column)**.

A pipeline describing the proposed method is available in Figure 1.

A detailed step-by-step guide can be downloaded as supplementary material in the corresponding section.

### Section 2

#### Data acquisition

High resolution 3D, T1-weighted spoiled gradient recovery MR images were acquired prior to electrode implantation. All subjects were scanned in a Phillips Achieva 1.5T magnet unit, with final in-plane isotropic resolution of 1 mm. (*TR*/*TE*/*TI* = 9.2/4.2/450 ms, matrix 256 × 256, bandwidth 31.2 kHz, FOV 256 × 256 mm, and 175 slices) in approximately 6–7 min. Another MRI was acquired within 24–72 h after implantation in order to evaluate clinical aspects, and location of IE. MRI of implanted patients has been shown to be safe, with respect to possible movement induced by electromagnetic fields and heating of electrodes (Davis et al., 1999; Carmichael et al., 2008).

For the automatic segmentation analysis we used pre-implant MR images to obtain adequate results and to avoid metallic deflection artifacts induced by the IEs.

CT scans for each patient were always performed immediately after placement of electrodes in order to visualize IEs contacts and as part of the clinical protocol for the evaluation of possible complications such as hematoma, contusions or subdural effusions that may require early treatment. CT images were acquired with an LSVCT GE, 64 detectors unit using 32cm FOV; 512 × 512 Matrix and 0.625 mm slice thickness with isotropic reconstruction volume at 1 mm.

#### Pre-processing

***Automatic segmentation and labeling of cerebral cortex, Extra-cerebral structures extraction and Pial surface 3D reconstruction***. Images from all patients were imported from the scanner using a DICOM receiver and transformed to Nifti (http://nifti.nimh.nih.gov) using the DCM2NII module from the free software MRIcron available at (http://www.mccauslandcenter.sc.edu/mricro/mricron/install.html).

We performed subject-specific cortical segmentation, skull stripping and extraction of 3D pial surfaces for further analysis using the Freesurfer image analysis suite, which is fully automatic and freely available for Unix and Macintosh platforms at (http://surfer.nmr.mgh.harvard.edu/). The entire process took about 20 h per patient in a quad-core processor, 2.0 Mhz Intel i5 PC, with 4 Gb of RAM memory.

Since we used pre-implant MRI; the process could at this instance be performed in advance of the surgical implantation to shorten valuable processing time.

The obtained parcellation of the cerebral cortex is based on gyral and sulcal structures (Fischl et al., 2004; Desikan et al., 2006). To achieve this automatic parcellation of the gray matter, the pial surfaces are inflated to obtain a sphere (Fischl et al., 1999a) and registered to a spherical atlas. This atlas uses individual cortical folding patterns to match cortical geometry across subjects (Fischl et al., 1999b).

Freesurfer's automatic surface extraction and parcellation procedures have been demonstrated to show good test-retest reliability across scanner manufacturers and across field strengths (Han et al., 2006; Reuter et al., 2012). Moreover this tool has been validated by measuring mean distance error maps for cortical labels on the brain surface and revealed that the mismatch is minimal (Desikan et al., 2006; Klein and Tourville, 2012). The errors were distributed almost entirely along the boundaries between the structures and on the magnitude of 1 mm when comparing manually segmentation made by experienced anatomist against automated labeling schemes.

***Registration of MRI with post implanted CT***. For each patient, we registered the high-resolution preoperative T1 MR image and post-implanted CT. We performed this only by using predefined options included in the BRAINSFit module (Johnson et al., 2007) within the 3D Slicer open source medical image analysis platform (http://www.slicer.org). In order to register images across modalities, we used a negated mutual information metric (MI) to quantify the similarity between the images and drive the registration algorithm (Viola and Wells, 1997). This choice is supported by several studies showing that MI performs well in the coregistration of MR and CT images (Studholme et al., 1996; Maes et al., 1997). We registered both images efficiently by using a hierarchical approach. The first step consisting on a rigid registration having 6° of freedom (DOF): 3 for 3D translation and 3 for 3D rotation. The second one on an Affine registration with 12 DOF: the 6 specified before plus 3 for 3D anisotropic scaling and 3 for 3D shear. No initial manual registration was used to perform this registration in BrainsFit module as we configured its first step to automatically compute the center of the head in both cases and calculate their alignment.

The brain shift produced by the implantation of depth electrodes is usually minimal once complications are properly excluded and considerable smaller than in other type of IE arrays that depends on extensive craniotomies. For that reason, as documented by others (Desai et al., 2010; Kubota et al., 2013; van Rooijen et al., 2013) we considered that post-implantation CT is sufficient for accurate electrode localization. We did not consider advantageous to apply a free-form of registration.

As a result of our registration process, we generated a new CT overlaid on top of the brain T1 MR image obtained from the preprocessing stage.

This semi-automatic procedure involves only the standardized basic selection of registration types; requiring minimal inputs from the user. This procedure assured that the CT images were aligned with all of the cerebral parcellations and 3D brain reconstructions provided by Freesurfer.

To avoid unexpected errors in the registration procedure, results were visually examined.

#### Identification of IEs using 3DSlicer software

The whole pre-processing stage was accomplished semi-automatically using predefined auto-analysis pipelines.

We determined the final exact position of each IE and individual contacts in all patients with respect to the underlying labeled cortex using a visualization procedure in the 3D Slicer platform.

To locate the anatomical structures adjacent to each electrode we visually inspected 2D multi-planar reconstructions of the co-registered CT and MR images. We used 3D surface renderings of the pial surfaces and the IEs, for a global overview of the final implantation process and also to assist in the localization of 2D multiplanar navigation.

Both approaches are described in the following sections.

***Multiplanar 2D Visualization***. Brain MR image of each patient along with its corresponding cortical labels and CT scan containing the IEs were jointly explored. This procedure, also used by other epilepsy centers (Desai et al., 2010; Gonzalez-Martinez et al., 2012; Kubota et al., 2013) allows one to identify the location of individual contacts on different anatomical cuts and its position according to the surrounding cortex.

The process involved setting an accurate density threshold and window level for the CT volumes containing the IEs along with appropriate adjustment of display and transparency options for the cortical parcellations. At that point each contact was manually selected in order to obtain additional information as anatomical cortical location and spatial coordinates that were displayed in the visualization panel.

Additionally; using the display function in SPM8 (freely available at http://www.fil.ion.ucl.ac.uk/spm/), we were able to manually select contacts that evidenced ictal onset activity providing individual MNI spatial coordinates; important to conduct group analysis (See Table 2). It was achieved registering and normalizing the previously aligned post-implant CT volume and T1 structural images to a standardized MNI template as described before (Ashburner and Friston, 2003) through the utility “Normalize (Estimate and Write)” in SPM8. (See more details in the supplementary material section and also at http://www.fil.ion.ucl.ac.uk/spm/doc/manual.pdf). Please note that the SPM software is a suite of MATLAB functions and subroutines from The MathWorks, Inc that requires commercial licensing.

**Table 2:** **Clinical information, neuroimaging, and exploration results for the four patients included in the proposed method**.

	**Patient 1**	**Patient 2**	**Patient 3**	**Patient 4**
Neuroimaging findings	Normal MRI	Bilateral HS	Normal MRI PET: left mesial hypometabolism	MRI: left temporal focal cortical dysplasia
vEEG and ictal semiology localization	Left posterior temporal-parietal or occipital	Temporal bilateral	Left temporal Left cingulum	Left lateral temporal Left mesial temporal
No. of electrodes	9	6	6	4
Implantation planning	2 L Supra and infra calcarine	2 R hippo	1 L hippo	1 L hippo
	1 L Heschl's Grs	3 L hippo	1 amygdala	3 L superior
	2 L posterior temporal and parietal	1 L heschl's Grs	2 frontal mesial	temporal-cortex
	2 L temporal and parietal language		2 frontal pole	
	2 L hippo anterior and posterior			
Electrodes post-implant position	2 L Supra and infra calcarine	2 R hippo	1 L hippo	1 L anterior hippocampus
	1 L heschl's Grs	1 L hippo	1 amygdala	1 L anterior sup-temporal
	2 L posterior temporal and parietal	2 L para hipp Ctx	2 frontal mesial	1 L Medialsup-temporal
	2 L temporal and parietal language	1 L heschl's Grs	2 frontal pole	1 L posterior sup-temporal
	2 L anterior hippo			
MNI coordinates for ictal onset IEs	(−7, −82, 8)	(−36, −28, −11) (−33, −27, −18)	(−25, −16, −21)	(−42, −8, −13)
Defined EZ	Left peri-calcarine Ctx.	L hippocampus L parahipp. Ctx	Left hippocampus	Left superior-temporal cortex
Postoperative engel evolution	Ia	Ia	Ib	Ia

***Brain 3D surface reconstructions***. For a global overview of the final implantation process, automatic 3D Pial reconstructions obtained from the freesurfer output were then overlaid with the IEs on the surface. We automatically implemented the Marching Cubes algorithm (Cline et al., 1987) to generate 3D representations of the electrodes by using the volume rendering technique (Drebin et al., 1988) which enables real-time 3D visualization and quantitative analysis of volumetric data. We accomplished the aforementioned steps by combining the use of “Model” and “Volume Render” modules for the Pial surface and IEs representations, respectively with adequate transparency and 3D display settings to the 3D visualization panel.

Thus the cortical surface renders clarified the deep trajectory of IEs and its relation with cortical structures. This procedure is also essential to assist operators during the 2D multi-planar navigation when the implantation planning is uncertain.

## Results

A neuro-radiologist processed the four patients of the first group according to this workflow. Two patients had normal MRI, one with left temporal cortical dysplasia and one showed bilateral hippocampal sclerosis. Twenty five depth electrodes were implanted and detected, ranging from 4 to 9, averaging 6 electrodes per patient (Table 2).

**Figure 2:**
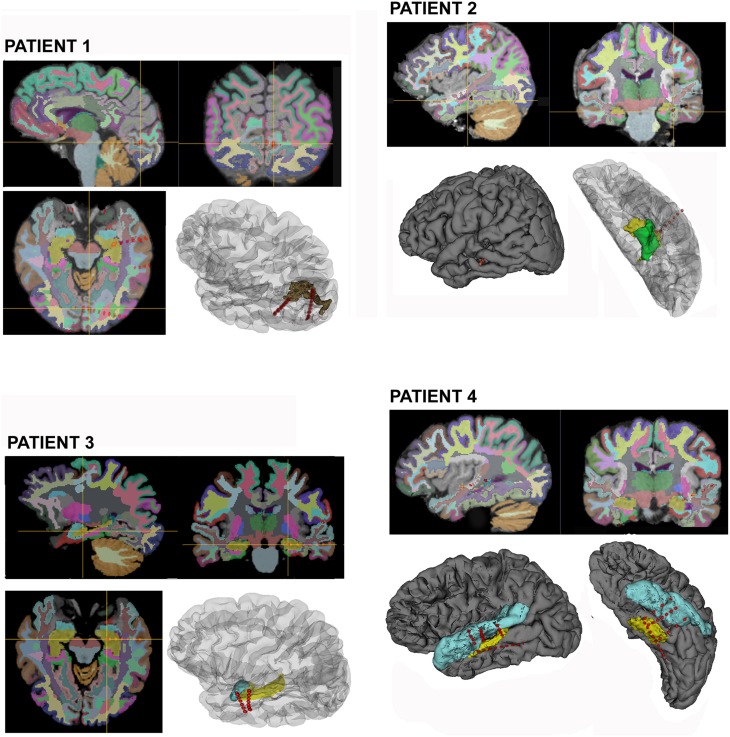
**Selected electrodes are displayed on 2D views for the four patients processed according to the proposed method, the cross bar highlight individual contacts that recorded ictal onset EEG discharge.** Cortical parcellations are overlaid and color coded according to Freesurfers lookup table. 3D surface renders from lateral and inferior views shows the electrodes trajectory and involved cortical structures. (Brown, Peri-calcarine cortex; Yellow, Hippocampus; Green, Para-hippocampal cortex; and Light Blue, Uncus and Superior temporal cortex).

The average time needed from implantation to the identification of the anatomical region involved with each IE's contact in this group was 10 h (6–24 h); for each patient. The contacts included in the EZ, are shown in (Figure 2) for all patients in this group.

Histopathology findings reported focal cortical dysplasia type IIb in two patients and hippocampal sclerosis in the other two.

The traditional visual analysis of IEs position performed on the control group was effective to localize every contact, but with an overall time range from 36 h to 3 days, restrained by the specialized personnel and the image quality available. The EZ was defined using this method in all cases for clinical decisions.

The follow-up (only 1 year) and good postoperative outcome (Engel 1) suggests that the definition of the EZ, eloquent areas and corresponding locations of IEs were correct in all patients from the first group. For the validation purpose trained operators correctly defined the anatomical localization of 5 ictal-onset contacts (See Materials and Methods) with an overall accuracy of 95%. Thus the anatomical localization of electrodes was correctly identified by two inexperienced operators in 19 of 20 tests, compared against the results of experts based on traditional visual analysis as a reference standard. Inter-raters agreement was excellent, between operator reliability was assessed using Kappa statistics (*k* = 0.875).

We also calculated the percent of agreement between sessions for each operator (5 tests per session) to estimate within operator reliability in the anatomical localization of ictal-onset contacts. The first operator concordantly identified the anatomical localization of each 5 contacts in both sessions. Concordance between sessions was achieved in the localization of 4 contacts for the second trained operator. Intra-observer overall agreement was 90%.

There have been no reports of adverse outcomes in epilepsy patients implanted at our center during MRI scanning following safety recommendations.

## Discussion

Several localization methods that involve qualitative estimates of electrodes locations based on visual assessment of RX, or CT, in addition to notes, sketches, and photographs acquired intra-operatively during the implantation have been proposed (Hill et al., 2000; Noordmans et al., 2001; Wellmer et al., 2002; Dalal et al., 2008). These estimates may have some limitations related to different issues including brain shift, lack of 3D representation or insufficient brain tissue contrast to precisely define anatomical regions.

More recent publications under very well controlled conditions describes effective approaches to identify IEs that may include considerable computational work, the use of specialized human resources or dedicated developments (Hermes et al., 2010; Dykstra et al., 2011; Yang et al., 2012; Pieters et al., 2013).

Appropriate techniques intended to localize IEs should guarantee high accuracy and precision but also the ability to be readily incorporated in clinical settings. Here we propose a method to localize IEs based on free software that has the potential to overcome the necessity of specialized personnel.

Inexperienced operators demonstrated good agreement and high accuracy defining the anatomical localization of the EZ in a limited sample of patients compared with experts.

It is important to underline that the automatic cortical segmentation provided by Freesurfer relies in the absence of structural anomalies evident on brain MRI. This process can only be applied using high resolution T1 MRI and this must be considered as a limitation for the proposed method.

Future developments will address the potential of our method to localize other type of electrode arrays as the use of subdural grids or strips, where a different approach is mandatory, constrained by brain shift associated with open neurosurgery (Schulze-Bonhage et al., 2002; Hunter et al., 2005; Kovalev et al., 2005).

One of the most important issues hindering rapid localization of IEs in our patients was time delay to post-implant neuroimaging but also the availability of qualified human resource.

Rapid access to specialized personnel is a common difficulty during chronic intracranial EEG recordings. Our method takes advantage of automatic anatomical segmentations and 3D visualization possibilities of well-established tools. This approach may assist epileptologists in the adequate and rapid localization of IEs if our findings are replicated in a larger number of patients. Further analysis will also provide the opportunity to obtain accurate quantitative estimates of the results.

### Conflict of interest statement

The authors declare that the research was conducted in the absence of any commercial or financial relationships that could be construed as a potential conflict of interest.
