# Supplementary material for: Rapid and efficient localization of depth electrodes and cortical labeling using free and open source medical software in epilepsy surgery candidates
Source: Front Neurosci. 2013 Dec 31;7:260. doi: 10.3389/fnins.2013.00260 (PMC3876273; doi:10.3389/fnins.2013.00260)
Supplement: Supplementary file 1 [file Presentation1.PPT]

## Slide 1
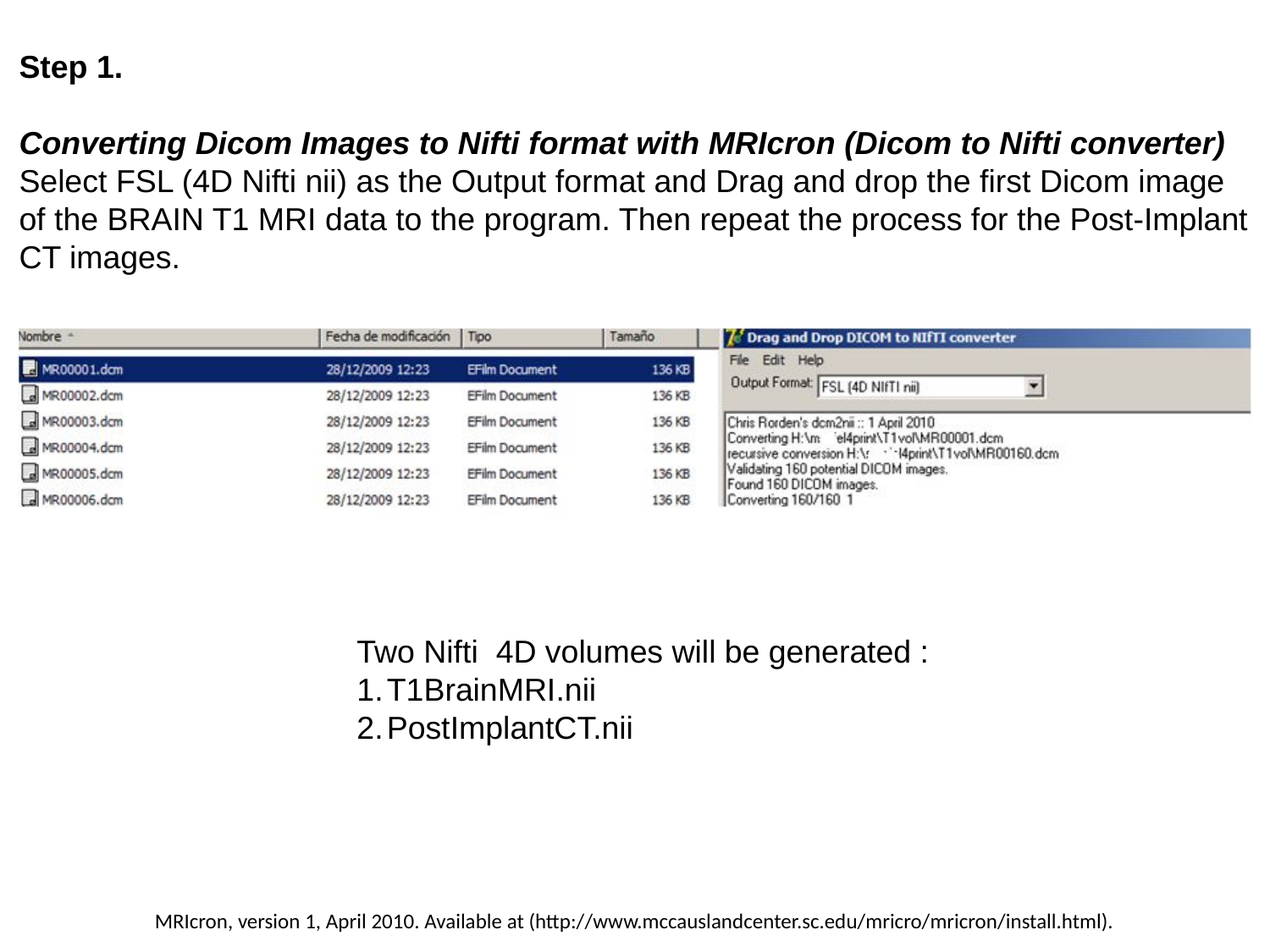

Step 1.
Converting Dicom Images to Nifti format with MRIcron (Dicom to Nifti converter)
Select FSL (4D Nifti nii) as the Output format and Drag and drop the first Dicom image
of the BRAIN T1 MRI data to the program. Then repeat the process for the Post-Implant
CT images.
Two Nifti 4D volumes will be generated :
T1BrainMRI.nii
PostImplantCT.nii
MRIcron, version 1, April 2010. Available at (http://www.mccauslandcenter.sc.edu/mricro/mricron/install.html).

## Slide 2
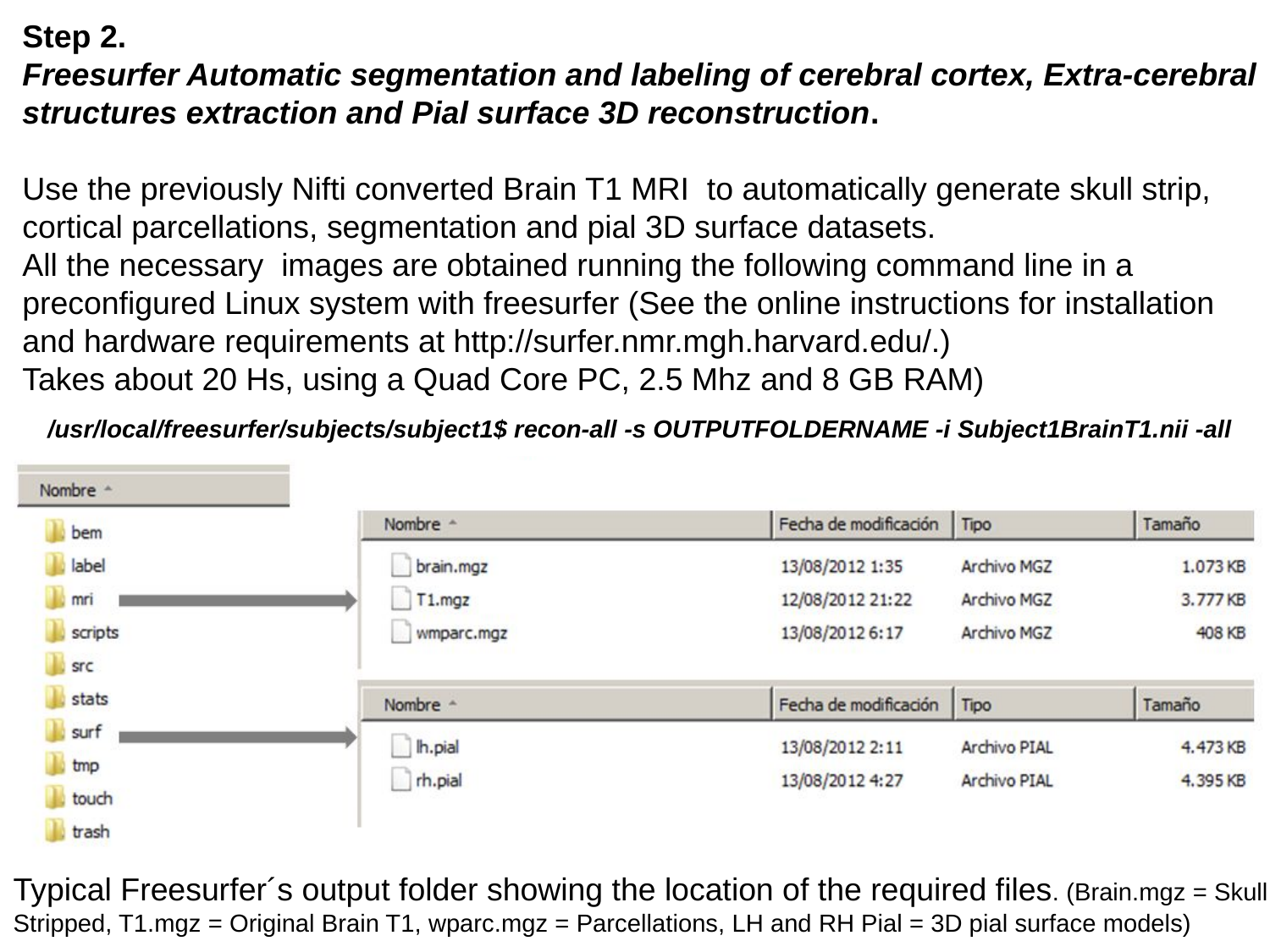

Step 2.
Freesurfer Automatic segmentation and labeling of cerebral cortex, Extra-cerebral
structures extraction and Pial surface 3D reconstruction.
Use the previously Nifti converted Brain T1 MRI to automatically generate skull strip,
cortical parcellations, segmentation and pial 3D surface datasets.
All the necessary images are obtained running the following command line in a
preconfigured Linux system with freesurfer (See the online instructions for installation
and hardware requirements at http://surfer.nmr.mgh.harvard.edu/.)
Takes about 20 Hs, using a Quad Core PC, 2.5 Mhz and 8 GB RAM)
/usr/local/freesurfer/subjects/subject1$ recon-all -s OUTPUTFOLDERNAME -i Subject1BrainT1.nii -all
Typical Freesurfer´s output folder showing the location of the required files. (Brain.mgz = Skull
Stripped, T1.mgz = Original Brain T1, wparc.mgz = Parcellations, LH and RH Pial = 3D pial surface models)

## Slide 3
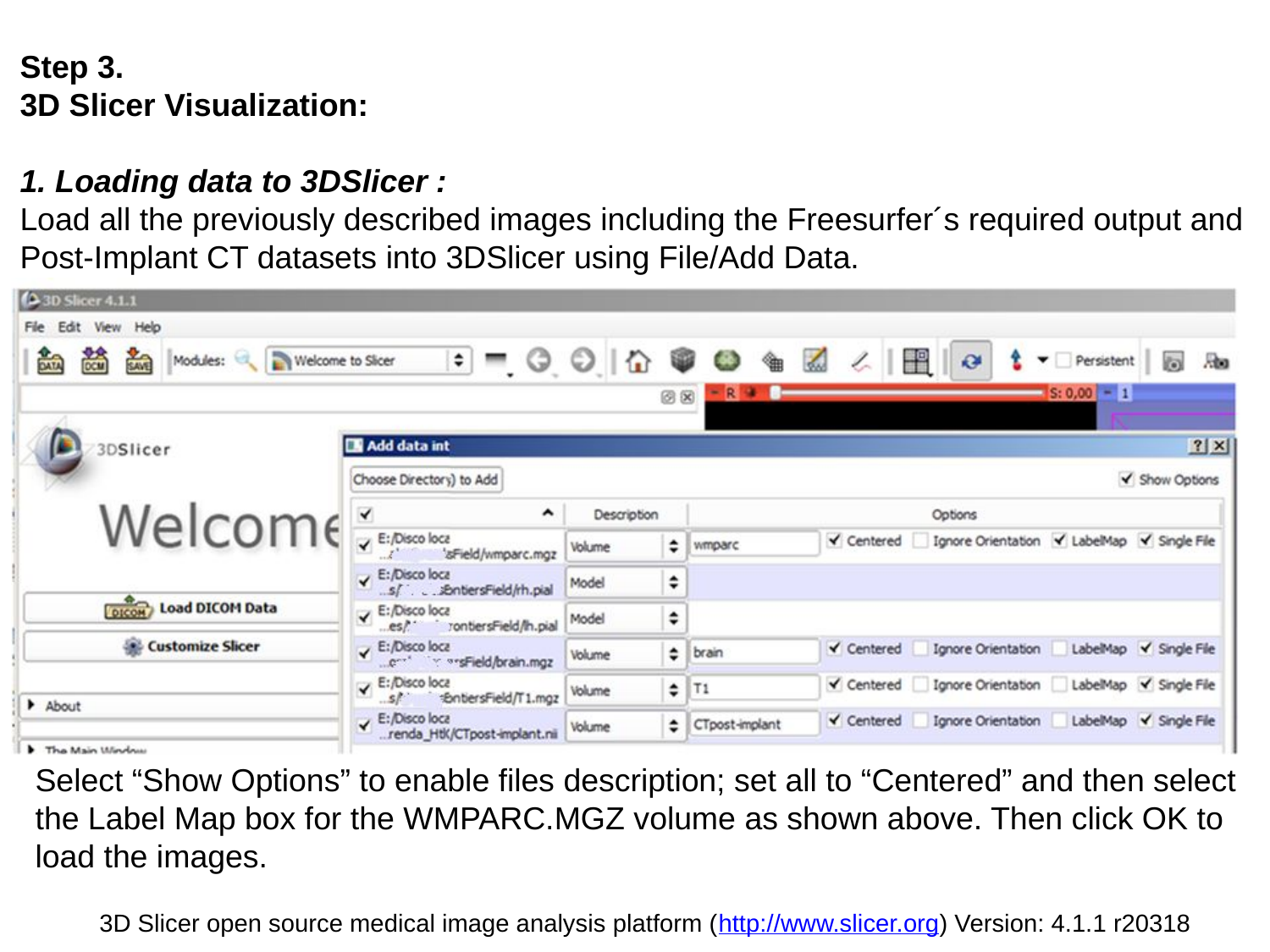

Step 3.
3D Slicer Visualization:
1. Loading data to 3DSlicer :
Load all the previously described images including the Freesurfer´s required output and
Post-Implant CT datasets into 3DSlicer using File/Add Data.
Select “Show Options” to enable files description; set all to “Centered” and then select
the Label Map box for the WMPARC.MGZ volume as shown above. Then click OK to
load the images.
3D Slicer open source medical image analysis platform (http://www.slicer.org) Version: 4.1.1 r20318

## Slide 4
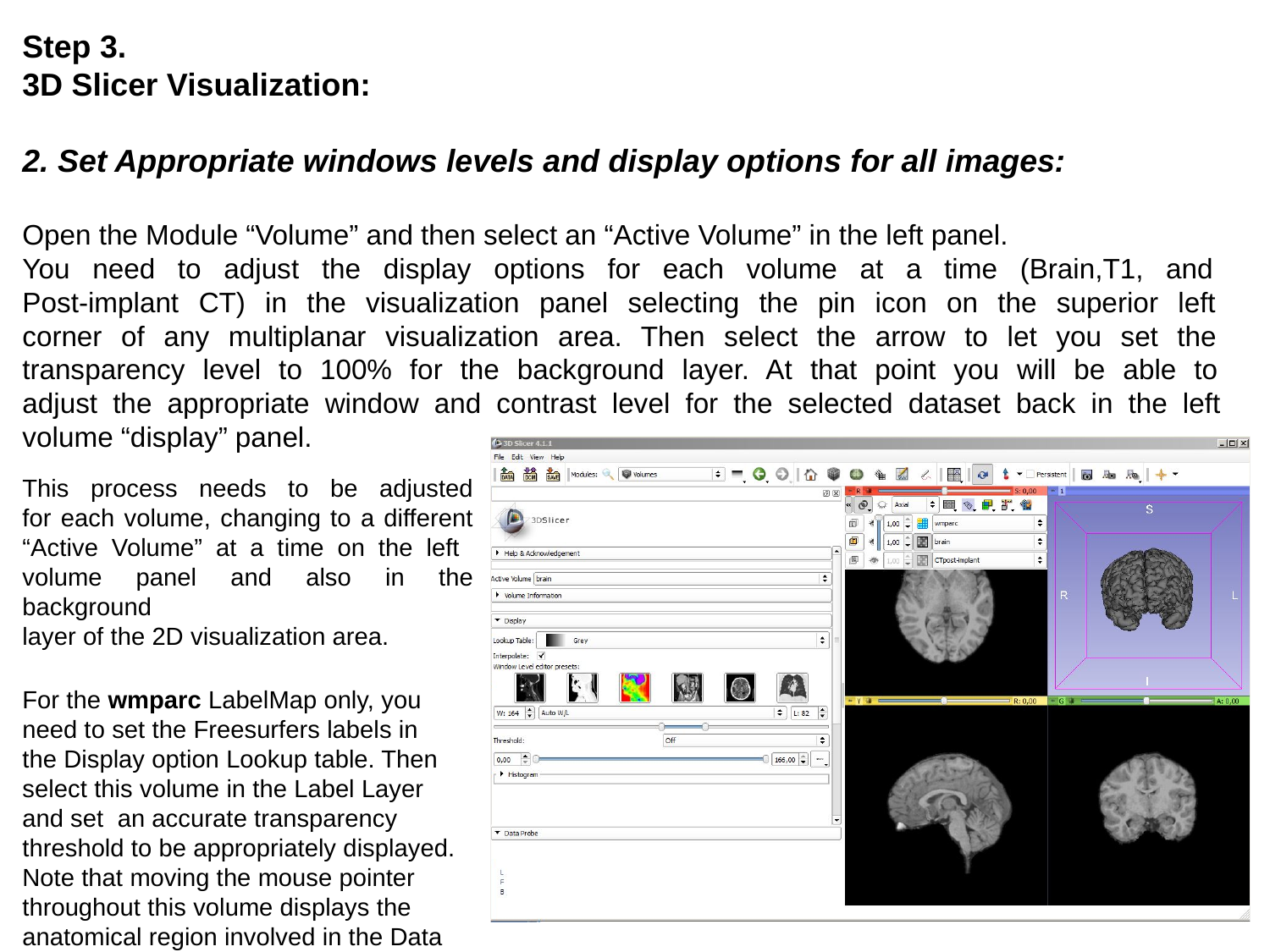

Step 3.
3D Slicer Visualization:
2. Set Appropriate windows levels and display options for all images:
Open the Module “Volume” and then select an “Active Volume” in the left panel.
You need to adjust the display options for each volume at a time (Brain,T1, and
Post-implant CT) in the visualization panel selecting the pin icon on the superior left
corner of any multiplanar visualization area. Then select the arrow to let you set the
transparency level to 100% for the background layer. At that point you will be able to
adjust the appropriate window and contrast level for the selected dataset back in the left
volume “display” panel.
This process needs to be adjusted
for each volume, changing to a different
“Active Volume” at a time on the left
volume panel and also in the background
layer of the 2D visualization area.
For the wmparc LabelMap only, you
need to set the Freesurfers labels in
the Display option Lookup table. Then
select this volume in the Label Layer and set an accurate transparency threshold to be appropriately displayed.
Note that moving the mouse pointer
throughout this volume displays the
anatomical region involved in the Data
Probe panel on the left.

## Slide 5
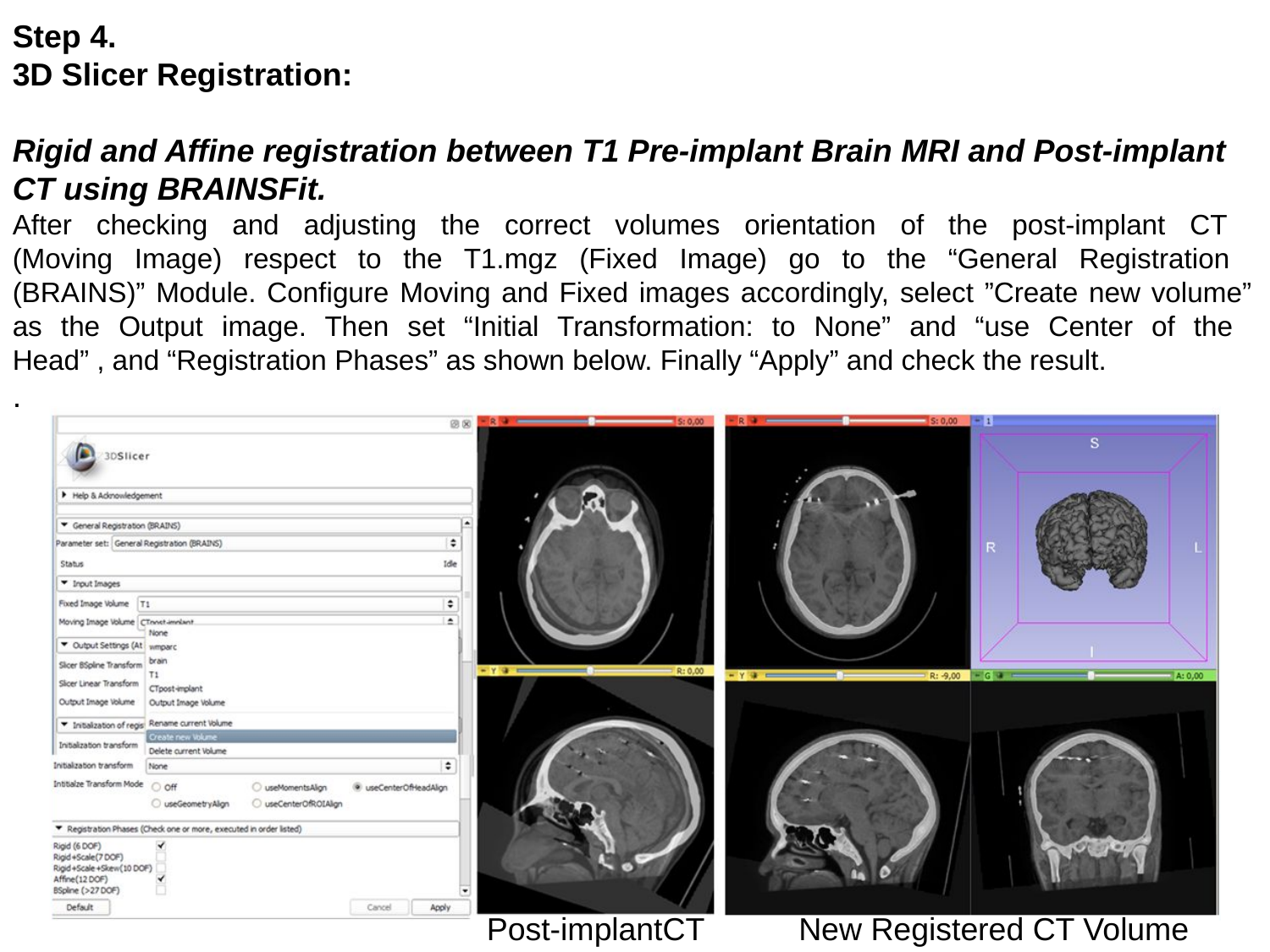

Step 4.
3D Slicer Registration:
Rigid and Affine registration between T1 Pre-implant Brain MRI and Post-implant
CT using BRAINSFit.
After checking and adjusting the correct volumes orientation of the post-implant CT
(Moving Image) respect to the T1.mgz (Fixed Image) go to the “General Registration
(BRAINS)” Module. Configure Moving and Fixed images accordingly, select ”Create new volume”
as the Output image. Then set “Initial Transformation: to None” and “use Center of the
Head” , and “Registration Phases” as shown below. Finally “Apply” and check the result.
.
Post-implantCT
New Registered CT Volume

## Slide 6
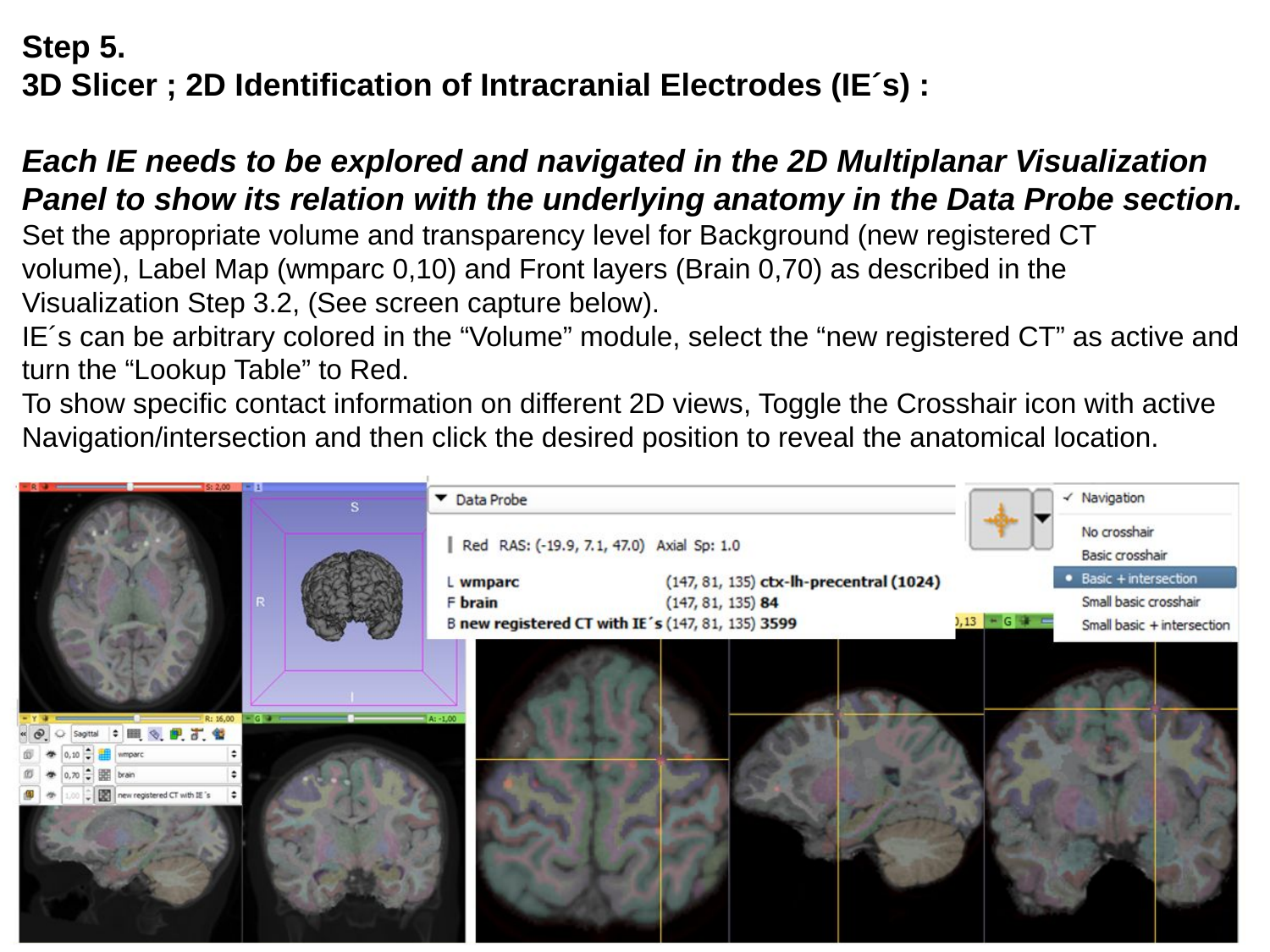

Step 5.
3D Slicer ; 2D Identification of Intracranial Electrodes (IE´s) :
Each IE needs to be explored and navigated in the 2D Multiplanar Visualization
Panel to show its relation with the underlying anatomy in the Data Probe section.
Set the appropriate volume and transparency level for Background (new registered CT
volume), Label Map (wmparc 0,10) and Front layers (Brain 0,70) as described in the
Visualization Step 3.2, (See screen capture below).
IE´s can be arbitrary colored in the “Volume” module, select the “new registered CT” as active and
turn the “Lookup Table” to Red.
To show specific contact information on different 2D views, Toggle the Crosshair icon with active
Navigation/intersection and then click the desired position to reveal the anatomical location.
.

## Slide 7
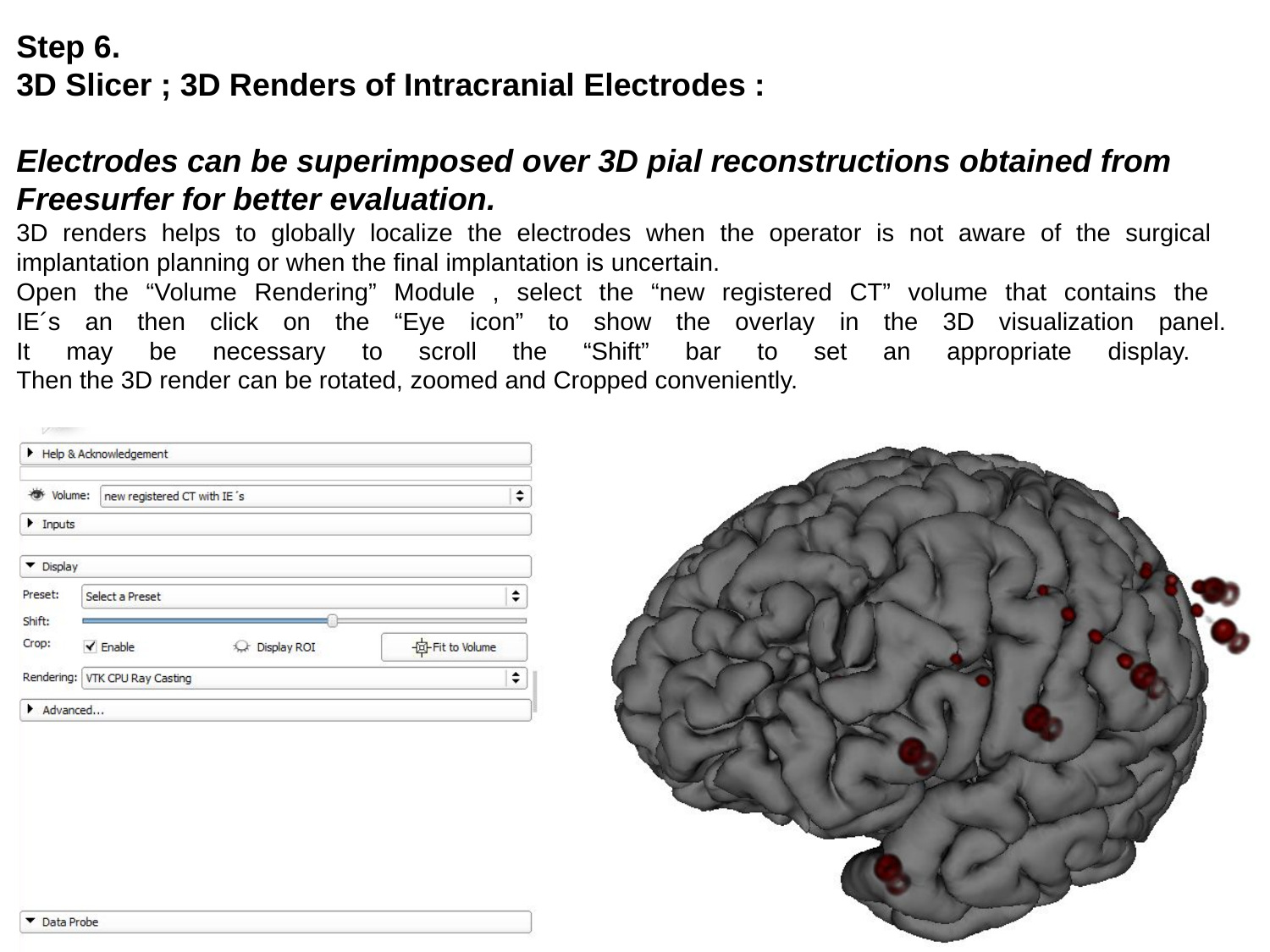

Step 6.
3D Slicer ; 3D Renders of Intracranial Electrodes :
Electrodes can be superimposed over 3D pial reconstructions obtained from
Freesurfer for better evaluation.
3D renders helps to globally localize the electrodes when the operator is not aware of the surgical
implantation planning or when the final implantation is uncertain.
Open the “Volume Rendering” Module , select the “new registered CT” volume that contains the
IE´s an then click on the “Eye icon” to show the overlay in the 3D visualization panel.
It may be necessary to scroll the “Shift” bar to set an appropriate display.
Then the 3D render can be rotated, zoomed and Cropped conveniently.

## Slide 8
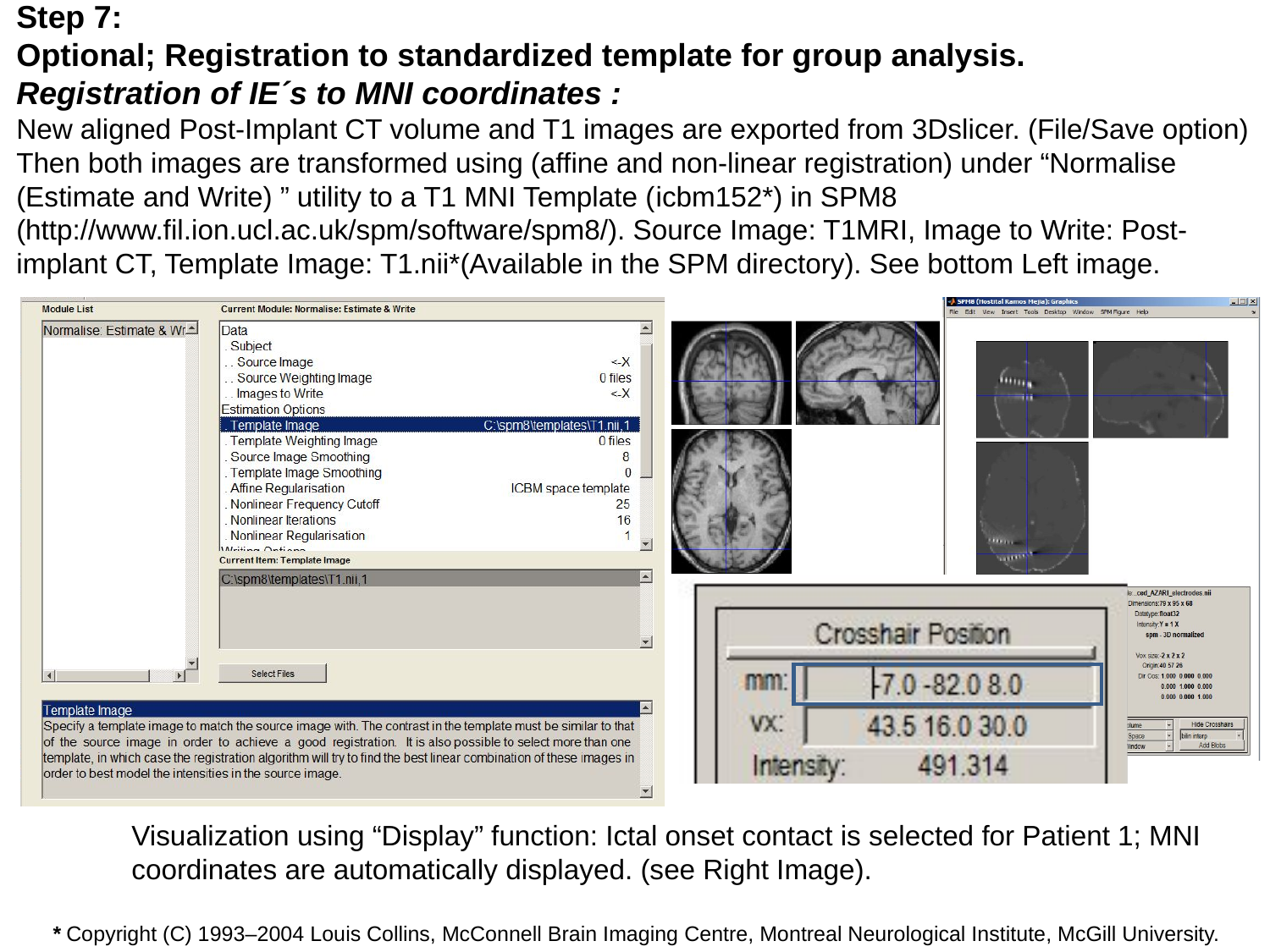

Step 7:
Optional; Registration to standardized template for group analysis.
Registration of IE´s to MNI coordinates :
New aligned Post-Implant CT volume and T1 images are exported from 3Dslicer. (File/Save option)
Then both images are transformed using (affine and non-linear registration) under “Normalise (Estimate and Write) ” utility to a T1 MNI Template (icbm152*) in SPM8 (http://www.fil.ion.ucl.ac.uk/spm/software/spm8/). Source Image: T1MRI, Image to Write: Post-implant CT, Template Image: T1.nii*(Available in the SPM directory). See bottom Left image.
Visualization using “Display” function: Ictal onset contact is selected for Patient 1; MNI
coordinates are automatically displayed. (see Right Image).
* Copyright (C) 1993–2004 Louis Collins, McConnell Brain Imaging Centre, Montreal Neurological Institute, McGill University.
